# On the Use of Acetic Acid in Preventing the Spread of Scarlatina

**Published:** 1850-09

**Authors:** J. W. Webster

**Affiliations:** Consulting Physician to the St. George’s and St. James’s Dispensary


*On the Use of Acetic Acid in Preventing the Spread of Scarlatina.* By Dr. J. Webster, Consulting Physician to the St. George's and St. James's Dispensary.—[Dr. Webster recommends frequent sponging of patients affected with scarlatina, with tepid vinegar and water, especially in the early stages, when the skin is hot or the pulse accelerated. He states that he has great confidence in the efficacy of this means in preventing the spread of the disease; and gives several cases in illustration of this.]—*London Journal, Dec.* 1849, *p.* 1295.

